# Rapidly Probing Antibacterial Activity of Graphene Oxide by Mass Spectrometry-based Metabolite Fingerprinting

**DOI:** 10.1038/srep28045

**Published:** 2016-06-16

**Authors:** Ning Zhang, Jian Hou, Suming Chen, Caiqiao Xiong, Huihui Liu, Yulong Jin, Jianing Wang, Qing He, Rui Zhao, Zongxiu Nie

**Affiliations:** 1 Beijing National Laboratory for Molecular Sciences, Key Laboratory of Analytical Chemistry for Living Biosystems, Institute of Chemistry Chinese Academy of Sciences, Beijing 100190, China; 2 University of Chinese Academy of Sciences, Beijing 100049, China; 3 National Center for Mass Spectrometry in Beijing, Beijing 100190, China

## Abstract

Application of nanomaterials as anti-bacteria agents has aroused great attention. To investigate the antibacterial activity and antibacterial mechanism of nanomaterials from a molecular perspective is important for efficient developing of nanomaterial antibiotics. In the current work, a new mass spectrometry-based method was established to investigate the bacterial cytotoxicity of graphene oxide (GO) by the metabolite fingerprinting of microbes. The mass spectra of extracted metabolites from two strains DH5α and ATCC25922 were obtained before and after the incubation with nanomaterials respectively. Then principal component analysis (PCA) of these spectra was performed to reveal the relationship between the metabolism disorder of microbes and bactericidal activity of GO. A parameter “D” obtained from PCA scores was proposed that is capable to quantitatively evaluate the antibacterial activity of GO in concentration and time-dependent experiments. Further annotation of the fingerprinting spectra shows the variabilities of important metabolites such as phosphatidylethanolamine, phosphatidylglycerol and glutathione. This metabolic perturbation of *E. coli* indicates cell membrane destruction and oxidative stress mechanisms for anti-bacteria activity of graphene oxide. It is anticipated that this mass spectrometry-based metabolite fingerprinting method will be applicable to other antibacterial nanomaterials and provide more clues as to their antibacterial mechanism at molecular level.

As the emergence of resistance to multiple antimicrobial agents in pathogenic bacteria has become a public health threat, the development of new antibacterial agents based on nanomaterials provides a valid strategy to combat multi-drug-resistant (MDR) pathogens^1^. Many nanomaterials have exhibited antibacterial activity such as gold^2^,^3^, silver^4^,^5^, ZnO^6^,^7^, TiO_2_^8^ nanoparticles and carbon-based nanomaterials including fullerene^9^, nanodiamonds^10^, carbon nanotube^11^,^12^, and graphene^13^,^14^,^15^. The establishment of analytical methods to rapidly evaluate (e.g. within hours) the antibacterial activities of these nanomaterials is essential for their discovery and optimization. Moreover, it is imperative to elucidate the antibacterial mechanisms for efficient designing of new nanomaterial antibiotics. Some nanomaterials such as nano-silver are reported to kill bacteria by blocking DNA replication, inhibiting protein synthesis, or inhibiting the formation of peptidoglycan and breaking down the cell wall, or via reactive oxygen species (ROS) production^16^,^17^,^18^. However, the molecular mechanisms of many antibacterial nanomaterials, especially carbon-based materials, are still under debate^19^,^20^,^21^.

Graphene-based nanomaterials have attracted much attention due to the strong antibacterial activity and less harm to humans or other mammals^13^,^22^. Graphene and graphene oxide nanowalls displayed antibiotic activity on both gram-positive bacteria (*S.aureus*) and gram-negative bacteria (*E. coli*), and the antibacterial mechanism of GO was explained to be membrane damage caused by the extremely sharp edges of the nanowalls^23^. By comparing the antibacterial activity of four types of graphite-based materials graphite, graphite oxide, graphene oxide (GO) and reduced GO towards *E. coli* using colony counting method, GO was confirmed to have the highest antibacterial activity^24^. Based on the bacterial morphology observation by electron microscopy and ROS detection evaluated by glutathione (GSH) content, it was proposed that both membrane and oxidative stress contribute to the bacterial cytotoxicity of graphite-based nanomaterials^24^. However, for the large-area monolayer graphene film, charge transfer from microbial membrane to graphene is considered as the antibacterial mechanism^14^. It was also found the antibacterial activity of GO is lateral-dimension dependent^25^, and cell entrapment mechanism was regarded as the main factor on antimicrobial activity of GO sheets in cell suspension^26^. Akhavan *et al.* proposed graphene oxide can be reduced by *E. coli* bacteria and formed bactericide graphene^27^. Lipid peroxidation was deemed to be another pathway that graphene nanosheets enhanced antibacterial efficiency^15^. Recently, it was ascertained experimentally and theoretically that graphene and graphene oxide can destructively extract phospholipids from *E. coli* membranes^13^, which offers a novel mechanism for cytotoxicity and antibacterial activity of graphene-based nanomaterials. These researches are helpful for the understanding the antibacterial mechanism of GO, nevertheless, the underlying molecular mechanisms are still not yet fully elucidated. Therefore, novel methods that could not only disclose the bacterial biophysicalchemical changes induced by nanomaterials^28^, but also pry into the molecular information of antibiotic invading^29^ are badly needed.

As the downstream outcome of cell machinery, small molecular metabolites can provide the phenotype of a biological system^30^. Changes of bacterial metabolites convey much valuable information on the interactions between nanomaterial antibiotics and bacteria. In metabolite profiling, mass spectrometry has taken an important place due to its high sensitivity and high throughput^31^,^32^. By analyzing the metabolic changes using mass spectrometry, many metabolites and their functions were discovered so that more detailed metabolic process can be investigated^33^,^34^. Additionally, it has been successfully applied for the detection of antibiotic resistance^35^,^36^,^37^. Mass spectrometry methods open new avenues in clinical and experimental microbiology study to investigate the antibacterial activity of nanomaterials from the molecular perspective^38^.

Herein, we established a mass spectrometry-based metabolite fingerprinting method for rapidly investigating the antibacterial activity of graphene oxide other than the time consuming colony counting method. By using this method, we attempted to explain its antibacterial mechanism by investigating the variability of metabolites. Briefly, the metabolite fingerprinting mass spectra were first obtained by matrix-assisted laser desorption/ionization time-of-flight mass spectrometry (MALDI-TOF MS), and then analyzed by principal components analysis (PCA). According to the PCA results, a parameter “D” which represents the distance between each experimental groups and the control group is introduced to evaluate the antibacterial activity of GO quantitatively. The result indicates that the bacterial cytotoxicity of GO will reach to a plateau even when the higher concentration and longer incubation time were used, which shows the same trend with results from traditional colony counting method. Furthermore, the relative antibacterial activities of GO and silver nanoparticles (AgNPs), hydroxylated fullerene or hexagonal boron nitride (h-BN) are compared by the parameter “D”. The PCA analysis also yields insights into the metabolic relationships between bacterial phenotypes before and after interacting with nanomaterials with plots of the principal component loadings. The results suggest the concentration changes of metabolites such as phospholipids and glutathione are closely related to the bacterial inactivity.

## Results

### MALDI-TOF MS Analysis of *E.coli* Extraction

**Figure 1:**
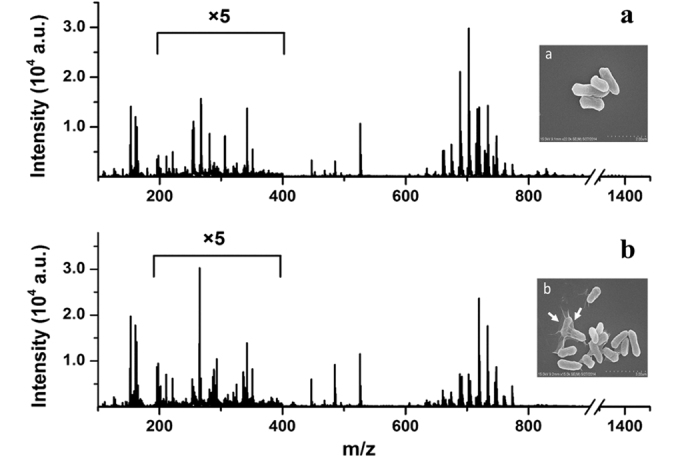
The mass spectra of *E. coli* extracts (**a**) without GO incubation (**b**) with GO (80 μg/mL) incubation for 2 h. Insets: the corresponding SEM images of *E. coli* and *E. coli* incubated with GO.

Nanomaterials as external stimuli cause the microorganism phenotype alteration. Graphene-based nanomaterials can fulfill the promises in the biomedical area as antibiotics or anticancer treatments^39^. In the current experiments, we aimed to observe the bacterial metabolism disturbance induced by Graphene Oxide (GO) through mass spectrometry. *E. coli* DH5α and *E. coli* ATCC 25922 were used as a model bacterium to evaluate the antibacterial activity of GO. In the experiment, the metabolites of *E. coli* were extracted by methanol^40^ and analyzed by MALDI-MS after 2h incubation with and without GO (80 μg/mL). From the typical metabolite fingerprinting mass spectra of *E. coli* DH5α in Fig. 1, peaks abundant in *m/z* 200–400 range were small metabolites like palmitoleic acid *etc.*, whereas the phospholipids of cytoplasmic membrane mostly appeared at *m/z* 600–800^41^. The fingerprinting mass spectra of ATCC 25922 was similar to DH5α. The mass spectra in Fig. 1 present that phenotypes of DH5α control group and GO incubated group were quite different, especially the patterns of peaks at *m/z* 600~800. Figure S3 represents the different areas of a certain peak in the two mass spectra, presenting the diversity of fingerprinting. The insets in Fig. 1 represent the corresponding SEM images of the control and GO incubated bacteria, which also displays the interaction between GO and the cell membrane of *E. coli*.

To identify the substances in mass spectra accurately, high resolution FT-ICR mass spectrometer (HR-MS) was utilized to determine the elementary composition of the detected ions. Combined with database searching (www.lipidmaps.org *and* www.ecmdb.ca), more than 50 substances have been identified (Table S1). These substances include small metabolites such as hypotamine (*m/z* 108.001), L-glutamic acid (*m/z* 146.018), palmitoleic acid (*m/z* 253.210), palmitic acid (*m/z* 255.217), heptadecenoic acid (*m/z* 267.218), vaccenic acid (*m/z* 281.184), glutathione (*m/z* 306.029) and dodecylbenzenesulfonic acid (*m/z* 325.122) *etc*. The most notable peak groups appearing at *m/z* between 600 and 800 were attributed to the lipids phosphatidylethanolamine (PE) and phosphatidylglycerol (PG) with different alkyl chains, which were identified by tandem mass spectra. PE and PG are the main building blocks of the *E. coli* membrane^42^,^43^. The identification of peaks further verified the distinct PE and PG patterns of *E. coli* at native state or incubated with GO, which indicates that the bacterial growth inhibition by GO may be related to the change of *E. coli* membrane.

### PCA Clustering of GO Incubated *E. coli*

**Figure 2:**
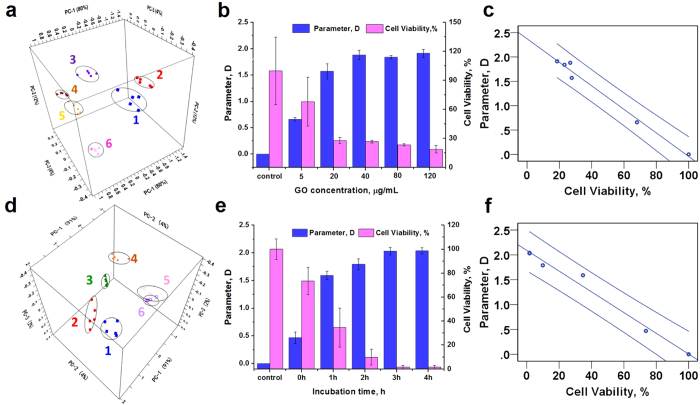
(**a**) The PCA results of *E. coli* DH5α incubated under different GO concentrations. 1 represents the control group and 2–6 principal components are from the samples incubated with different GO concentrations at 5, 20, 40, 80 and 120 μg/mL for 2 h. (**d**) The PCA results of time experiment. 1 represents the control group. 2–6 represent principal components obtained from the data incubated with GO (80 μg/mL) at different time: 0, 1, 2, 3 and 4 h. (**b**,**e**) show the relationship between GO concentration or incubation time and the parameter D as well as the cell viability rate of *E. coli* DH5α. (**c**,**f**) present the bivariate analysis results, and a significant negative correlation between the parameter D and cell viability rate were obtained in concentration experiment (Person’s correlation coefficient: −0.99, *p* < 0.01 in c) and time experiment (Person’s correlation coefficient: −0.99, *p* < 0.01 in f).

**Figure 3:**
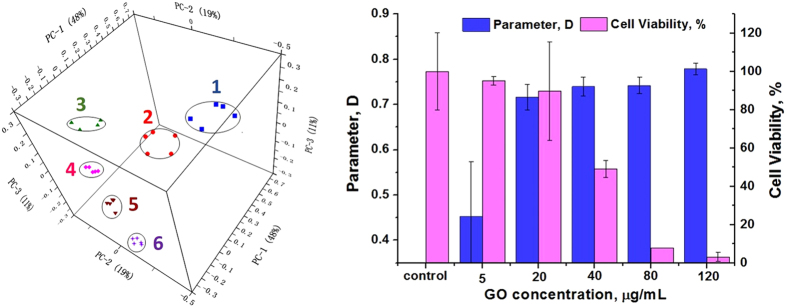
(**a**) The PCA results of ATCC 25922 metabolites under different GO concentrations incubation. 1 represents the control group with GO and 2–6 principal components are from the ATCC 25922 samples incubated with GO concentrations at 5, 20, 40, 80 and 120 μg/mL for 2 h respectively. (**b)** shows the relationship between GO concentration and the parameter D as well as the cell viability rate of ATCC 25922 calculated by colony method.

PCA utilizes orthogonal transformation of correlated metabolite profiles into linearly uncorrelated PCs to separate varied samples^44^. In this work, PCA was operated based on the metabolite fingerprinting mass spectra of *E. coli* DH5α and ATCC 19522 incubated with gradient GO concentrations (0, 5, 20, 40, 80 and 120 μg/mL). The results show that the phenotypes of *E. coli* DH5α under different GO concentrations are clustered into a single quadrant. There is little overlap of the cluster with each other in 3D PCA space (Fig. 2a). In Fig. 2a, each spot represents one mass spectrum and each principal component contains five spots. Besides, the cluster analysis (CA) results can categorize the different groups of microbes, indicating a good reproducibility of the experiment (Figure S6). For ATCC 25922 analysis, the PCA result is consistent with DH5α. The phenotypes of ATCC 25922 are categorized by the GO concentration. There is little overlap of the clusters with each other in 3D PCA space (Fig. 3a).

### Analysis of GO antibacterial activity quantitatively

A parameter “D” is proposed to represent the distance between the experimental groups and the control group in PCA space. We assume that as the value of D becomes larger, the greater the difference of phenotypes between the experimental group and the control group, which suggests better antibacterial activity of nanomaterials. The relationship between D and GO concentration in Fig. 2 indicates that with the increase of the GO concentration, the value of D increases and finally reaches a plateau. It is interesting that the tendency of D to predict antibacterial activity is consistent with *E. coli* DH5α viability calculated by colony method. We further performed the bivariate analysis of “D” calculated by PCA scores and the cell viability obtained by colony counting method, and got a Pearson correlation coefficient of −0.99. The result indicates a significant negative correlation of these two factors (*p* < 0.01) (Fig. 2c). Similarly, it is observed in Fig. 2d that the phenotypes of *E. coli* incubated with GO incubation at different time are separated from each other. With the increase of incubation time, the value of D increases gradually and the *E. coli* DH5α viability decreases (Fig. 2e). Again, the bivariate analysis of PCA distance and viability of *E. coli* DH5α shows a significant negative correlation of these two factors (*p* < 0.01) with Pearson correlation coefficient of −0.99 (Fig. 2f). For ATCC 25922, Fig. 3b shows the value of D increases with GO concentration. The tendency of D versus GO concentration is similar to the viability versus GO concentration by colony method.

To verify the feasibility of the method to other species, *Klebsiella pneumonia*, another gram-negative bacteria, was selected to incubate with GO at different concentration. Although the metabolite fingerprinting of the *Klebsiella pneumonia* are quite different from that of *E. coli*, it presents the same tendency that with the increase of GO incubation concentration, the cell viability of *Klebsiella pneumonia* decreases and the related D value increases (Figure S7).

The antibacterial activity of another antibacterial nanomaterial, AgNPs (2 nm), was also investigated by the proposed method. As a common antibacterial agent, AgNPs has been proven to have highly effective antibiotic ability and has been employed extensively to fight infections^45^. The similar results were observed in that as the AgNPs concentration goes up, the D value gradually increases, and the cell viability of *E. coli* DH5α decreases as obtained by colony counting method (Figure S8). These results further confirmed our finding that D value could be used as an indicator of antibacterial activity of nanomaterials. This method can rapidly detect and characterize bacterial responses to antibiotics. We speculate the relative antibacterial activity of nanomaterials can also be evaluated by comparing the D value for each antibacterial agent.

**Figure 4:**
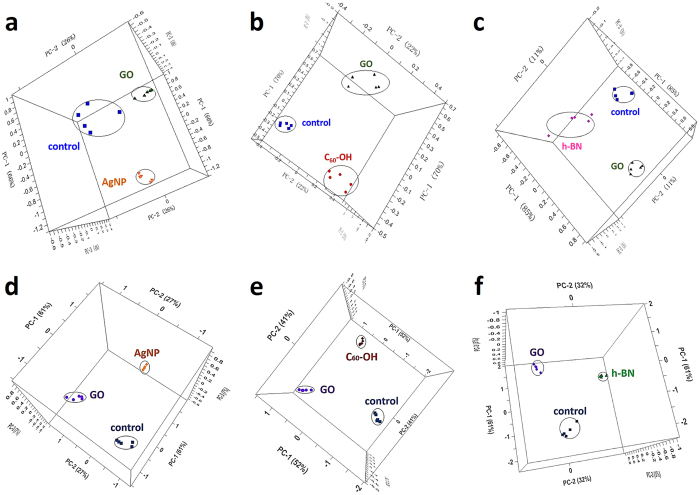
(**a**) The three principal components in PCA scores plot represent the *E coli.* DH5α control group (◾), GO group (▴) and AgNPs (▾) group (5 μg/mL, 2 h). (**b**) The three principal components in PCA scores plot represent the *E coli.* DH5α control group (◾), GO group (▴) and hydroxylated fullerene group (⦁) (80 μg/mL 2 h). (**c**) The three principal components in PCA scores plot represent the control group (▾), and the GO groups (▴) and hexagonal boron nitride (♦) group (80 μg/mL, 2 h). (**d–f**) The three principal components in PCA scores plot of ATCC 25922 represent the control group (◾), the GO group (▴) and AgNPs (▾) group (5 μg/mL, 2 h), hydroxylated fullerene group (⦁) (80 μg/mL 2 h) and hexagonal boron nitride (♦) incubation (80 μg/mL, 2 h), respectively.

To validate this assumption, we first compared the antibacterial activity of GO and AgNPs by the same method performed with *E. coli* DH5α. The PCA result is shown in Fig. 4a. It is obvious that the distance of the AgNPs incubated group (D = 1.47) is farther than GO (D = 0.95) incubated group from the control group, which means AgNPs have stronger sterilization ability than GO under the same experimental condition (5 μg/mL, 2 h). The result was confirmed by colony counting method. Another carbon nanomaterial, hydroxylated fullerene, was selected to be compared with GO at 80 μg/mL incubation concentration for 2 h. It was reported that at high concentration, fullerene shows antibacterial activity by inducing changes in the structural and elastic properties of the lipid bilayer^46^. Figure 4b presents that the antibacterial activity of GO is higher than hydroxylated fullerene by calculating the parameter D from PCA scores. The colony counting method also shows that the fullerene incubated cells were more viable than GO incubated ones. Furthermore, hexagonal boron nitride (h-BN), another layered materials was chosen to explore the shape effects on toxicity of nanomaterial. The PCA result of GO and h-BN incubated *E. coli* DH5α metabolites in Fig. 4c demonstrates that the antibacterial activity of GO is higher than h-BN with D = 0.92 for GO and D = 0.50 for h-BN, respectively. For ATCC 25922 stains, D value of AgNPs (2.25) is larger than D value of GO (1.70) (Fig. 4d). Figure 4e and f show the antibacterial abilities of hydroxylated fullerene and h-BN are also slightly lower than GO at given conditions.

### Antibacterial Mechanism Investigation

**Figure 5:**
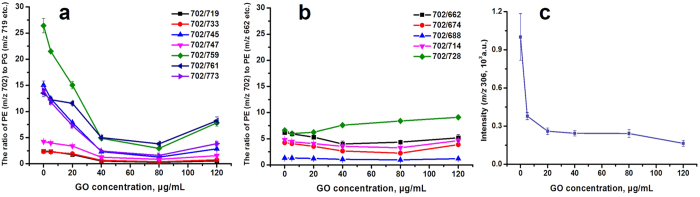
(**a**) Trend line of peak intensity ratio of PE (*m/z* 702)/PG (*m/z* 719, 733, 745, 747, 759, 761, 773) versus the GO incubation concentrations. (**b**) The change of peak intensity ratio of PE (*m/z* 702)/PE (*m/z* 662, 674, 688, 714, 728) with the change of GO incubation concentrations in the same coordinate in Fig. 4a. (**c**) The change of peak intensity of GSH at *m/z* 306 with the GO incubation concentrations.

The PCA loadings plot of fingerprinting mass spectra from gradient GO concentration experiments is shown in Figure S9 and it demonstrates distinct profiles of metabolite alterations after GO invading *E. coli*. From the results shown in Figure S9, it can be deduced that the substances at *m/z* 688, 702, 719, 733 *etc.* which belonging to phospholipids play important roles in the antibacterial process of GO. As confirmed by previous mass spectral analysis, the representative signals at *m/z* 688, 702, 719 and 733 *etc.* which correspond to PE and PG, are the main building blocks of *E. coli* membrane. We plotted the intensity ratio of PE (*m/z* 702)/PG (*m/z* 719, 733, 745, 747, 759, 761, 773) versus the concentration of GO. As shown in Fig. 5a, with the increase of GO concentration, the intensity ratio of PE/PG decreases gradually. However, in Fig. 5b, the ratio of PE (*m/z* 702)/PE (*m/z* 662, 674, 688, 714, 728) barely changes with the GO increase of incubation concentration. It is reasonable to deduce that interacting with the membrane lipids is one of the pathways by which GO displays its antibacterial activity. It was demonstrated that GO induced degradation of *E. coli* cell membranes including two types of molecular mechanisms: i) severe insertion and cutting membrane; ii) destructive extraction of lipid molecules^13^. Although the contact and tearing up the membrane of *E. coli* by GO could be observed by SEM (Fig. 1 insets), the change of substances cannot be elucidated. However, the mass spectrometry-based metabolite fingerprinting analysis conducted here provide direct molecular information about the bacteria phenotype in bacterial growth inhibition.

We further analyzed the supernatant solution which possibly encompassed the phospholipids that were extracted by GO from *E. coli* membrane. The mass spectra in Figure S11 confirmed that the PE at *m/z* 702, which attributed to the most abundant membrane components, does exist in the 80 μg/mL GO incubated solution (Figure S11b), but can be barely found in the control solution (Figure S11a). Some of the phospholipids were not quantified due to their ultra-low concentrations in the supernatant. Besides, we found that the intensity of another kind of phospholipid phosphatidic acid (PA 30:2) begins to grow then goes down with the increase of GO concentration, and the variation of it was shown in Figure S12.

Apart from phospholipids, some other substances including palmitoleic acid (*m/z* 253), palmitic acid (*m/z* 255), heptadecenoic acid (*m/z* 267), vaccenic acid (*m/z* 281) and dodecylbenzenesulfonic acid (*m/z* 325) have been found to change during GO incubation with *E. coli* (80 μg/mL), which has been list in Table S2. When the intensity ratio is more than 1, it means the signal intensities of these substances increase. Another important substance is glutathione (GSH, *m/z* 306), which is shown in Fig. 5c. It is found that the peak intensity of GSH goes down with the GO concentration changing from 0 to 120 μg/mL.

## Discussion

Though carbon-based nanomaterials such as graphene oxide are potential antibiotics to fight MDR bacteria infections, the antibacterial activity and mechanism is far from explicit in a molecular view. In the current work, we provide a novel mass spectrometry based method to determine the antibacterial activities of GO by the change in metabolites for the first time. Principal component analysis of metabolite fingerprinting of *E. coli* DH5α and ATCC 25922 after incubation with GO at different conditions shows metabolic disturbance of these bacteria (Figs 2a,d and 3a,b). The differences of PCA were represented using a distance parameter “D”. The results Fig. 2c and f show potent evidence to confirm “D” is closely related to the survival condition of the microbe, and there is a significant negative correlation between the parameter “D” and the bacterial viability. And the results confirmed the antibacterial activity of GO is concentration- and time-dependent. The PCA results and tendency of D values versus GO concentration when analyzing the fingerprinting of ATCC 25922 are identical with DH5α. Thus, through the value of D, which is calculated from the PCA scores, the antibacterial activity of GO nanosheets can be evaluated. These results testify that the PCA analysis of metabolites fingerprinting can be used to investigate the antibacterial activity of antibiotics.

From the above results, it can be concluded that as the value of D becomes larger, the greater the difference of phenotypes between the experimental group and the control group, suggesting higher antibacterial activity of the nanomaterial and greater effectiveness as an antibiotic. By comparing the value of D calculated from different antibacterial agents, the relative antibacterial activity of these antibacterial agents can be evaluated. According to this principle, we compared the antibacterial activity of GO and AgNPs, hydroxylated fullerene or h-BN by this method. The results of *E. coli* DH5α and ATCC 25922 both indicate that at the same experimental conditions, the antibacterial activity of GO is lower than AgNPs, but higher than hydroxylated fullerene and h-BN, which was also confirmed by colony counting method. Hydroxylated fullerene and GO are both carbon-based nanomaterials, but with different structures. h-BN and GO both have similar layer structure (Figure S2), but with different element composition. It shows both morphology and elemental composition are closely related to the antibacterial activity of carbon-based nanomaterials. Therefore, it is believed that the method can be applied to evaluate the relative antibacterial activity of different materials in scientific research and practical application.

**Figure 6:**
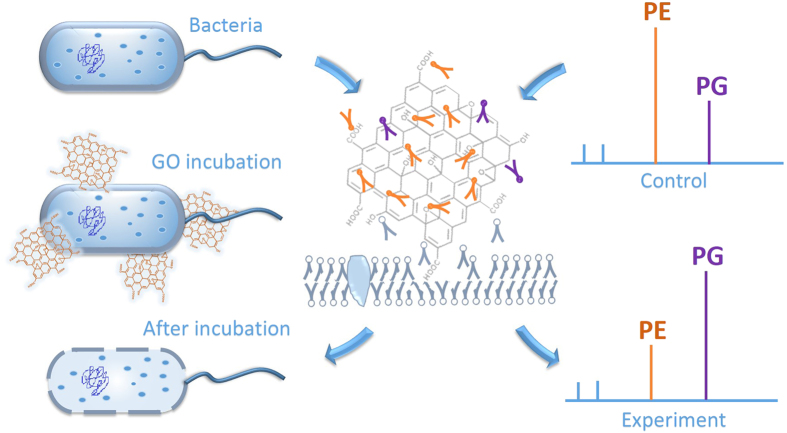
Schematic illustration of the antibacterial process of graphene oxide monitored by mass spectrometry.

The plots of principal component loadings show that most substances contributing to the cluster separations are the phosphatidylethanolamine (PE) and phosphatidylglycerol (PG) (Figure S9), which consists of a hydrophilic head group ethylenediamine or glycerol and hydrophobic tails respectively. The two kinds of phospholipids participate in many important life processes of *E. coli* including metabolism of membrane and translocation of protein^43^. In *E. coli* membrane, 70% of phospholipid composition is PE and the cell will stop growth and division when the content declines to 30–40%^47^. Different from PE, as an anionic phospholipid, PG was reported to be dispensable in *E. coli*^48^,^49^. Herein, the *zeta* potential of GO is negative (Figure S1), and except for hydrophobic interaction^13^ of tails with GO, the electrostatic interaction between materials and anionic phospholipid in *E. coli* membrane should be considered for the interaction between GO and bacterial membrane. It is not unreasonable that the electrostatic repulsion between GO and the polar head of PG makes the interactions between GO and PG weaker than that between GO and PE. Therefore, according to the destructive lipid extraction theory^13^, PE will be extracted out from the membrane more than PG (the process shown in Fig. 6). It is in agreement with the change of relative intensity of PE and PG observed from the mass spectra of extracted metabolite. Our experimental results further show the relative intensity of reserved PE and PG goes down with increasing GO concentration (Fig. 5).

Another variable phospholipid is PA, which is considered as the precursor that substitutes for PG in certain essential biological functions in *E. coli*^36^. Due to the low concentration, only one kind of PA (30:2) (*m/z* 671) was identified in our experiments. As shown in Figure S12, the increase of PA can be explained from less PG loss from the membrane and the phospholipid precursor PA accumulates and substitutes for it at low GO concentration. When GO concentration is high enough to kill most *E. coli* cells, more extracted metabolites including PA were lost from the cells, thus the peak intensity decreases subsequently.

Besides, GSH is another important metabolite during the antibacterial process of GO. As reported, GSH is an important antioxidant in bacterial cell, reflecting the information about reactive oxygen species (ROS) such as free radicals and peroxides. The decrease of glutathione means that the ROS in the cells increase during the GO incubation process (Fig. 5c), which hints the accumulation of ROS is also an explanation to the antibacterial mechanism of GO nanosheets. However, according to the PCA loadings plot (Figure S9), the contribution of glutathione at *m/z* 306 to the group separation is smaller than that of phospholipids. Different from GO, the loadings plot of AgNPs antibacterial experiment (Figure S10) shows the weights for small molecular are larger than that for phospholipids, which suggests the main antibacterial mechanisms of AgNPs and GO are different.

In order to make clear the detail antibacterial procedure of antibacterial agents at the molecular level, further investigation is necessary. For instance, it is worth to investigate the relationship between those molecules in Table S2 with the phospholipid metabolism of bacteria, which would be a clue to find the particular pathways influenced by antibacterial agents so that more confirmed antibacterial mechanisms are proposed.

Using MALDI-TOF MS combined with PCA analysis, the changes of metabolites at the molecular level were probed and the antibacterial procedure and mechanism of GO nanomaterial may be inferred. Firstly, the dispersed GO nanosheets contact with *E. coli* cells and cause stress upon *E. coli* membrane. Then, by interacting with membrane phospholipids, the *E. coli* membrane is damaged accompanied by increase in reactive oxygen species, finally promoting the death of bacteria.

## Conclusion

We have demonstrated a method for evaluating the antibacterial activity of graphene oxide and investigated its antibacterial mechanism in a molecular perspective using mass spectrometry-based metabolite fingerprinting of microbes. Typical metabolites including glutathione, phosphatidylethanolamine and phosphatidylglycerol are identified. By analyzing PCA scores, the parameter “D” between experimental groups and control group was confirmed to be closely related to the antibacterial activity of GO nanosheets. By monitoring changes of some metabolites, our method puts forward one way to reveal the membrane destruction and oxidative stress mechanisms of GO nanosheets. Specifically, the changes of PE and PG suggest that GO nanosheets contact and interact with membrane lipids and the decrease of glutathione implies the increase of ROS. We believe that the proposed method will be particularly useful in rapid antibiotic activity tests. Its application opens a new way to research the interaction of nanomaterials and microbes.

## Methods

### Chemicals and Materials

The HPLC grade methanol was purchased from Thermo Fisher. Culture media components including trptone, yeast extract, and agar were purchased from Oxoid. Sodium chloride was purchased from Sinopharm Chemical Reagent Beijing Co., Ltd. Nano-silver solution (1000 ppm, 2 nm) was purchased from Shanghai huzheng nanotechnology Co., Ltd. Graphene oxide (GO), hexagonal boron nitride (h-BN) and hydroxylated fullerene were purchased from Nanjing XFNANO Materials Tech Co., Ltd. N-(1-naphthyl) ethylenediamine dihydrochloride (NEDC) was purchased from Sigma-Aldrich. Water used in all experiments was deionized using a Milli-Q ultrapure water purification system (Merck, Ltd., USA). The purchased GO and h-BN were dispersed in sterile saline water (2 mg/mL) as stock solution by 2 h ultrasonic oscillation. The original nano-silver solution was diluted to 2 mg/mL for the experiment. The hydroxylated fullerene can be directly dispersed in sterile saline water and the concentration of the stock solution is also 2 mg/mL. The graphene oxide was characterized by TEM, AFM, XPS and Raman and the results were shown in Figure S1, from which we can obtained that the thickness of GO nanosheet is about 0.8 nm, suggesting its single layer structure. For the h-BN, the thickness of the nanoplate is about 25 nm (Figure S2).

### Cell preparation

*E. coli* DH5α and ATCC 25922 were grown in LB (Luria-Bertani) medium at 37 °C with 150 rpm shaking speed, and harvested in the stationary phase, whose final optical density of 0.5 at 600 nm (OD_600nm_ = 0.5). Cultures were centrifuged at 9000 rcf for 10 min to pellet cells and washed with sterile water, then the pellets were resuspended in sterile saline water for incubation experiments with cell sample containing 10^5^ to 10^6^ CFU/mL. For the bacteria Klebsiella Pneumonia, the treatment process is the same as that of *E. coli* DH5α.

### Cell incubation

500 μL microbe cells above were used to incubate with fresh GO dispersions in sterile saline water with a final volume of 1.5 mL at 37 °C under 250 rpm shaking speed, and the same conditions for other nanomaterials including AgNPs, hydroxylated fullerene and h-BN nanoplates. For the GO concentration experiment, the incubation concentration of GO are 0, 5, 20, 40, 80 and 120 μg/mL for 2 h, respectively. For the GO time experiment, the incubation time is 0 h, 1 h, 2 h, 3 h and 4 h with 80 μg/mL GO nanosheets. For the experiment of AgNPs, *E. coli* incubated with AgNPs at different concentration (0, 2.5, 5, 10 and 20 μg/mL) for 2 h. For the antibacterial activity comparison experiments, the incubation concentraions of hydroxylated and h-BN are both 80 μg/mL. For the experiment using Klebsiella Pneumonia, the incubation concentration of GO are 0, 5, 20, 40 and 80 μg/mL for 2 h, respectively. The colony counting method was utilized to evaluate the loss of bacterial cell viability. Briefly, series of 10-fold cell dilutions (100 μL each) were spread onto LB plates, and left to grow overnight at 37 °C. Colonies were counted and compared with those on the control plates to calculate the cell viability. The bacteria without GO nanosheets was used as the control, and all treatments were prepared in triplicate.

It should be noted that in the comparison experiments, the concentration of different antibaterial nanomaterials should be the same. For AgNPs, the cell viability rate of bacteria will below 20% when incubated with 20 μg/mL for 2 h, and the change of the metabolite fingerprinting will be the greatest. Even incubated with AgNPs at 5 μg/mL for 2 h, the change of metabolite fingerprinting is evident. If the incubation concentration is more than 20 μg/mL, more bacterial metabolites would lose, which is not beneficial for mass spectrometry analysis. However, for GO, when incubated with GO below 20 μg/mL for 2 h, the change of the metabolites is relatively unobvious. Therefore, to guarantee the same concentration condition, for the comparision of GO and AgNPs, the results of a small incubation concentration (5 μg/mL for both GO and AgNPs) were selected to compare their antibacterial activity. It is anticipated that the similar result will be obtained if 20 μg/mL incubation concentration for GO and AgNPs was usedAs to the other nanomaterials including hBN and hydroxylated fullerene, under small incubation concentration (<40 μg/mL), the cell viability is still high and the change of metabolites is minor which is difficult to differentiate from the control group in PCA analysis. To obtain a significant difference of D value for easily understanding our proposed method, the result of 80 μg/mL concentration was finally selected to compare the antibacterial activities of GO and hydroxylated fullerene or h-BN.

### Metabolite extraction

After incubation, the bacteria were centrifuged at 9000 rcf to pellet cells, and washed three times with sterile water. Then 100 μL HPLC methanol was added in pellet cells and ultrasonic oscillation for 10 min to extract the metabolites of bacteria. After centrifugation, the supernatant was obtained and condensed using nitrogen blowing instrument and re-dissolved in 10 μL HPLC methanol for MALDI-TOF MS analysis. Then a series of mass spectra were obtained, and to dig more important information about the antibacterial activity of GO, PCA was operated based on the metabolite fingerprinting mass spectra.

### Mass spectrometry analysis

MALDI-TOF MS analysis was performed on a Bruker Ultraflextreme MALDI-TOF/TOF mass spectrometer (Bruker Daltonics, Germany). The mass spectrum was acquired with the sum of 500 laser shots at 1000 Hz. A mixture of mono-, di-, tri-, tetra- and penta-saccharide (*m/z* 215, 377, 539, 701, 864) was used for mass calibration and the matrix is NEDC (20 mg/mL, water/ethanol 2:1). The ratio of sample and matrix is 3:1. High resolution mass spectrometry identification was conducted on Solarix (9.4 T, Bruker Daltonics, Bremen, Germany) equipped with ESI/MALDI dual ion source including SmartbeamII laser. Mass spectrum was first calibrated using Na_m_(CH_3_COO)_n_^−^ clusters in the negative mode. An error inferior to 0.5 ppm was systematically reached. MS/MS analysis of identified substances were performed both on MALDI-TOF/TOF and LTQ mass spectrometer (Thermo Scientific).

### SEM experiment

To assess the cell morphological characteristics after exposure to GO nanosheets, the cells were fixed by exposure to 2.5% glutaraldehyde solution for 30 min, and then dehydrated by using a series of concentrations of ethanol (e.g., 30, 50, 70, 80, 90, 95 and 100% ethanol solutions, each for 10 min). Afterwards, the cells were coated with platium and observed by SEM (HITACHI S-4800 Field Emission Scanning Electron Microscopy).

### Principal components analysis (PCA)

PCA was carried out using The Unscrambler X software with leverage correction type. The data used for PCA analysis were obtained from the mass spectra of bacterial extraction samples, including that with and without nanomaterials at different incubation concentration and time. Prior to PCA, the *m/z* values in each mass spectrum were normalized by divided the max value, and each mass spectrum was considered as a dependent variable. From the results of PCA analysis, we can obtain a PCA scores plot and a PCA loadings plot, which exhibits the weights for each original variable when calculating the principal components. For current profiles, the original variables are the values of *m/z* in the mass spectra. The loadings plot was used here to pick out the most significant metabolites that contribute to the PCA variations

### Distance determination of PCA clusters

The PCA space location of each spot is determined by the values of PC_1_, PC_2_ and PC_3_ scores. To evaluate the relationship among GO incubated *E. coli* quantitatively, a parameter, “D”, representing the distance between each experimental groups and the control group, is introduced. Specifically, D is defined as follows:


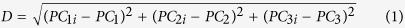


in the formula, PC_1_, PC_2_ and PC_3_ are the arithmetic averages of PC scores of the control group. PC_1i_, PC_2i_ and PC_3i_ are the PCA scores of the experimental groups at respective GO affected conditions.

### Supporting Information

Characterization of Graphene Oxide by TEM, AFM, XPS and Raman etc. PCA loading plots and clustering analysis of mass spectrometry data; list of identified metabolites in *E. coli*. This material is available free of charge via the Internet.

## Additional Information

**How to cite this article**: Zhang, N. *et al.* Rapidly Probing Antibacterial Activity of Graphene Oxide by Mass Spectrometry-based Metabolite Fingerprinting. *Sci. Rep.* **6**, 28045; doi: 10.1038/srep28045 (2016).

## Supplementary Material

Supplementary Information
