# Supplementary material for: Rapidly Probing Antibacterial Activity of Graphene Oxide by Mass Spectrometry-based Metabolite Fingerprinting
Source: Sci Rep. 2016 Jun 16;6:28045. doi: 10.1038/srep28045 (PMC4910068; doi:10.1038/srep28045)
Supplement: Supplementary Information [file srep28045-s1.doc]

Rapidly Probing Antibacterial Activity of Graphene Oxide by mass spectrometry-based Metabolite Fingerprinting

Ning Zhang^1,2^‡, Jian Hou^1,2^‡, Suming Chen^1^, Caiqiao Xiong^1^, Huihui Liu^1^, Yulong Jin^1^, Jianing Wang^1^, Qing He^1,2^, Rui Zhao^1,2^ and Zongxiu Nie^1,2,3^*

### *1. Beijing National Laboratory for Molecular Sciences, Key Laboratory of Analytical Chemistry for Living Biosystems, Institute of Chemistry Chinese Academy of Sciences, Beijing 100190, China;*

*2.University of Chinese Academy of Sciences,Beijing 100049, China;*

*3.* *National Center for Mass Spectrometry in Beijing, Beijing 100190, China.*

‡ Equal contribution to this work.

* Corresponding author:

Dr. Zongxiu Nie; Email: [znie@iccas.ac.cn](mailto:znie@iccas.ac.cn)

Key Laboratory of Analytical Chemistry for Living Biosystems

Institute of Chemistry Chinese Academy of Sciences

No. 2 Zhongguancun North 1^st^ St, Beijing 100190, China

Phone: +86 -10-62652123; Fax: +86 -10-82612849

**Characterization of graphene oxide.**


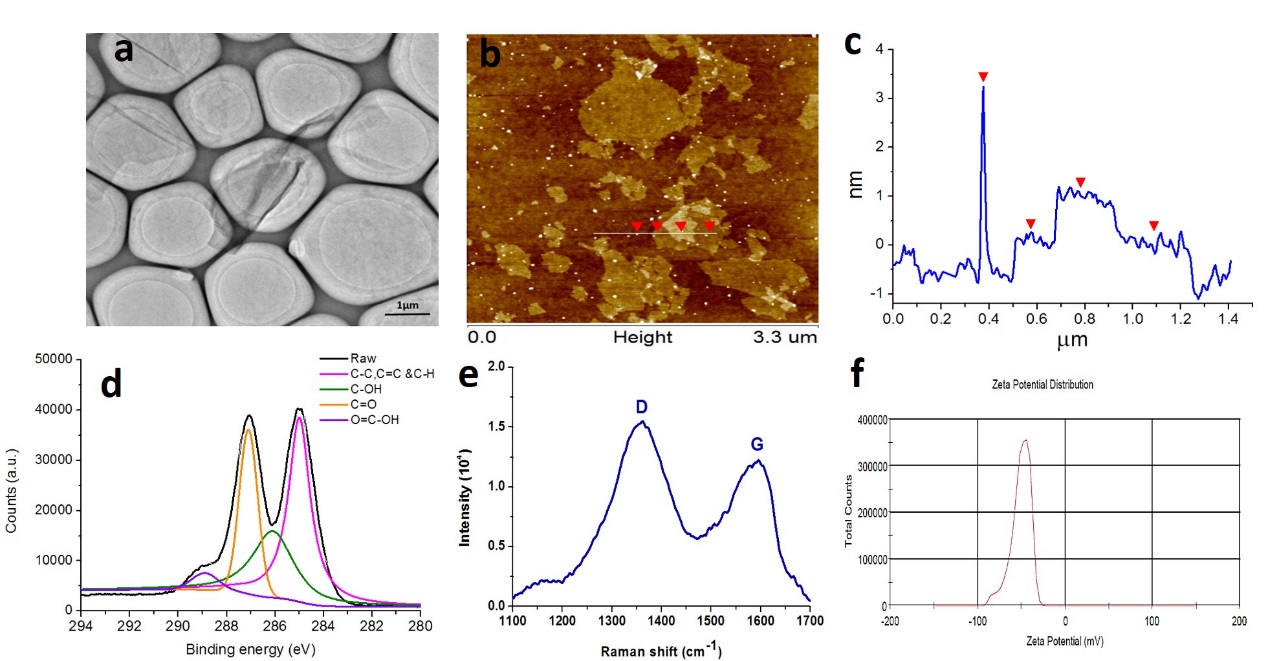


**Figure S1**. The characterization of graphene oxide by (a) TEM, (b) and (c) AFM, (d) XPS spectra, (e) Raman spectroscopy and (f) Zeta potential. The results the show that the the thickness of GO used in the experiment is about 0.8nm, which suggests single layer structure. The XPS spectrum of the GO exhibits the characteristic peaks of carbon skeleton, C-OH, C=O and O=C-OH at 285, 286.2, 287.1 and 288.9 eV, respectively. Raman spectrum of GO shows the presence of a G band at about 1580cm^-1^ and a D band at about 1350cm^-1^, which are assinged to the graphitized structure and local defects/disorders particularly at the edges of grahene oxide. The *zeta* potential of GO in solution is negtive.


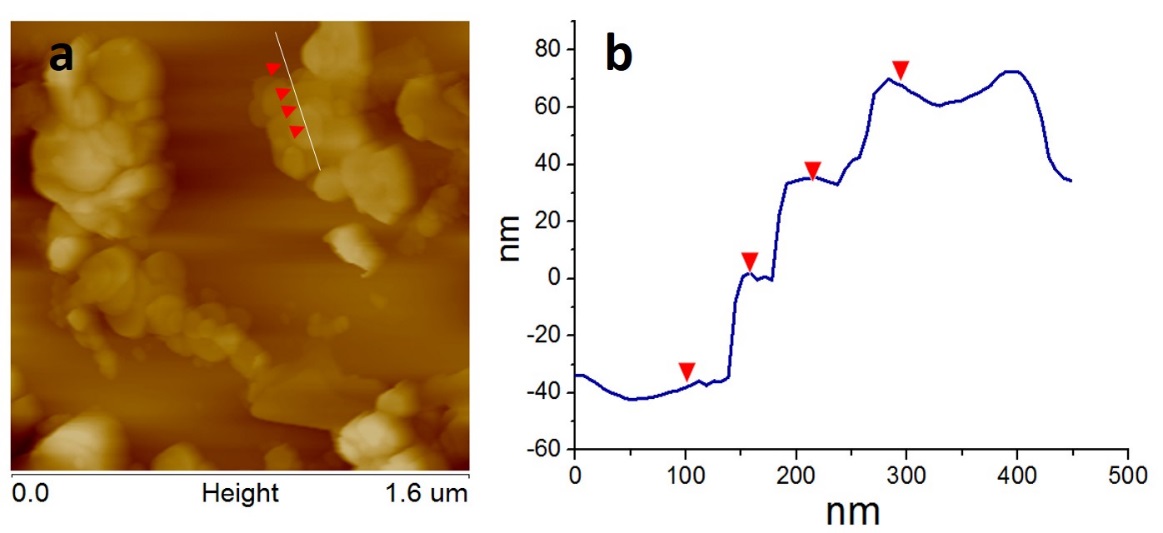


**Figure S2**. The characterization of h-BN by AFM. The result shows that the thickness of h-BN used in the expriment is about 25 nm.


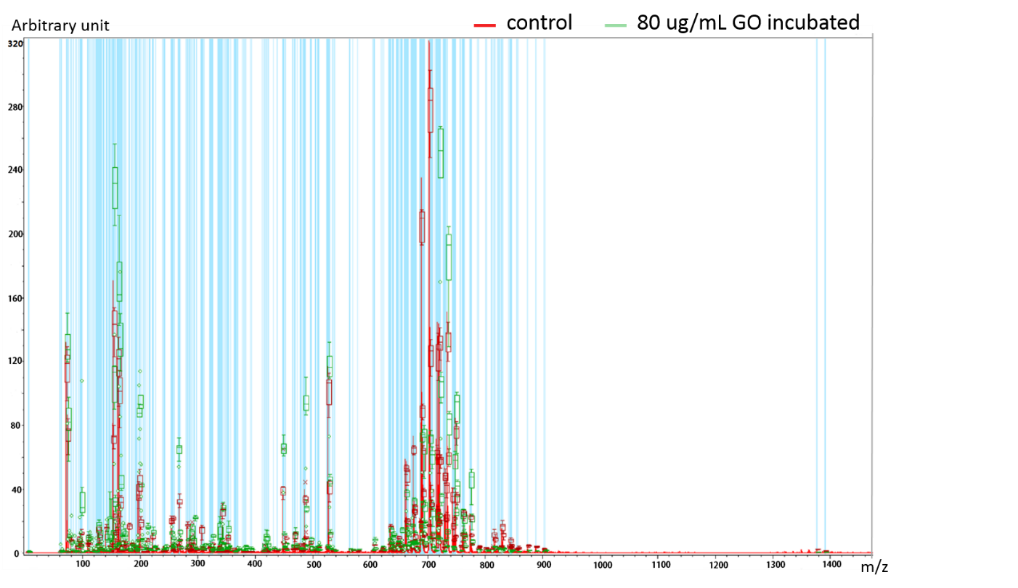


**Figure S3**. The per-class box for the intensity of the two mass spectra (control and 80μg/mL GO incubated). The box indicates the 25%-quartile and the 75%-quartile and the horizontal intersection denotes the median. The box plots give a graphic representation of homogeneity of the areas of a certain peak in the spectra of one class.

**Identification of substances.** We take the signal at *m/z* 719.565 in TOF as an example to illustrate the identification process. The precise mass obtained from HR-MS is 719.48747, which presumed as phosphatidylglycerol (32:1) by searching the lipid database ([*www.lipidmaps.org*](../../../../D:%5Cmass%20spectrometry%5C组会资料%5C张宁%5CGO%20Ecoli%5CE.coli%5Cwww.lipidmaps.org)) and *E.coli* database ([*www.ecmdb.ca*](http://www.ecmdb.ca/)) with mass deviation only 0.8ppm. In addition, the MS/MS spectrum in Figure S3 shows the precursor ion has fragment ions at *m/z* 437.23 (neutral loss of 18:1 chain), 281.25 (C18H35COO^-^) and227.20 ([Glycerophosphoglycerol-H-H_2_O]^-^), which is agree with PG (18:1/14:0) in database. Consequently, the signal is confirmed as PG (18:1/14:0). For some lipids, we obtained their MS/MS spectrum by MALDI TOF/TOF (Figure S4).


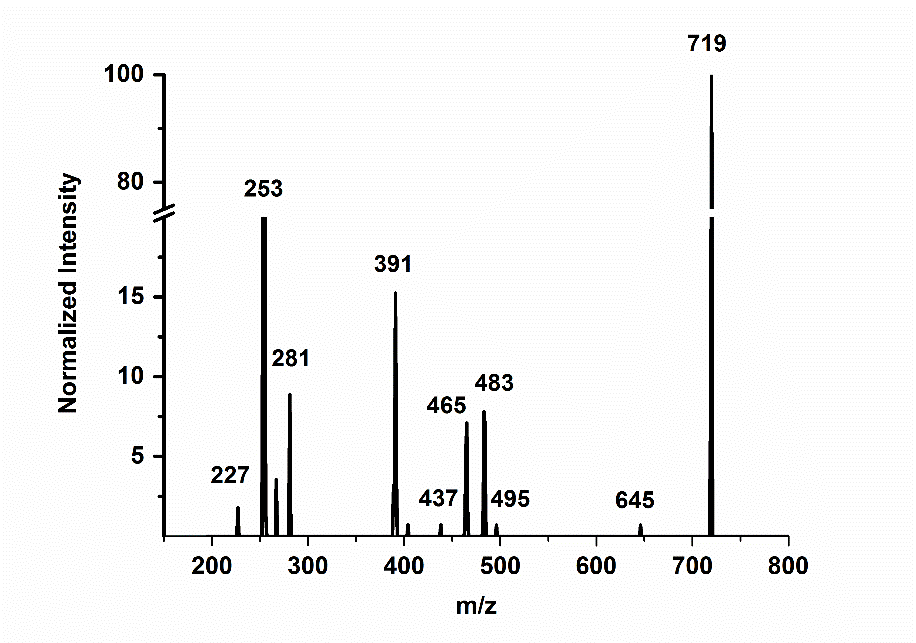


**Figure S4**. MS/MS spectra of selected ions 719 by LTQ ion trap.


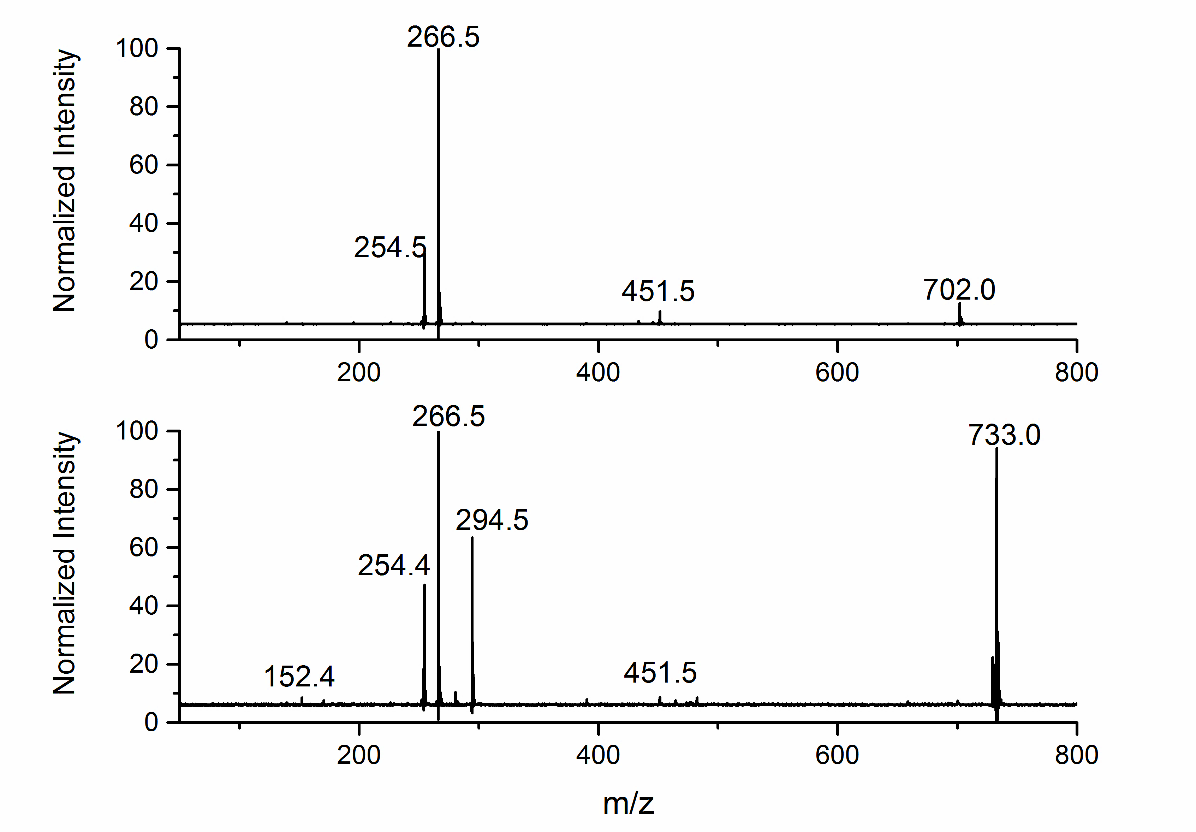


**Figure S5**. Tandem MS analysis of m/z 702 in a) and m/z 733 in b) by MALDI TOF/TOF.


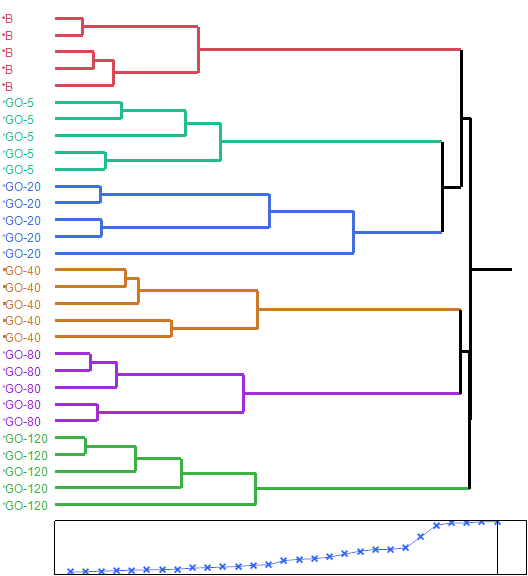


**Figure S6.** The cluster analysis of concentration and time experiments, which indicates the data obtained from MALDI-TOF MS possess good reproducibility. B represent the sample without GO incubation, GO-5, GO-20, GO-40, GO-80 and GO-120 represent the samples incubated with GO at 5, 20, 40, 80 and 120μg/mL.


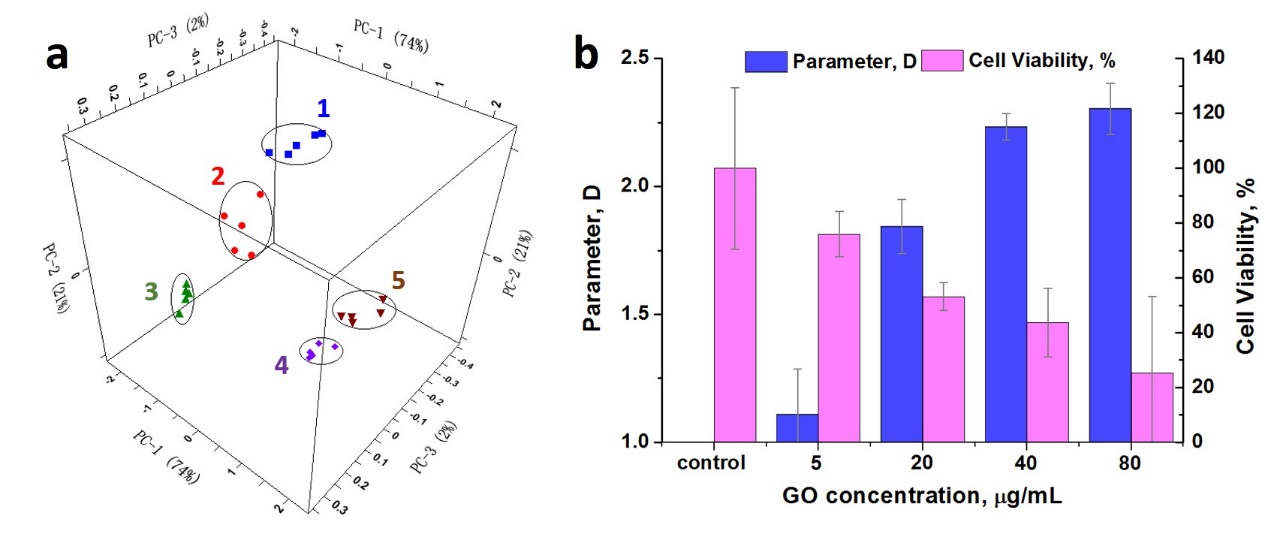


**Figure S7.** **a.** The PCA results of GO antibacterial experiment for the bacteria Klebsiella pneumonia. 1-5 principal components are from the sample incubated with different GO concentration at 0, 5, 20, 40 and 80μg/mL for 2h. Figure **b** shows the relationship between GO concentration and parameter D as well as the cell viability rates of Klebsiella Pneumonia.


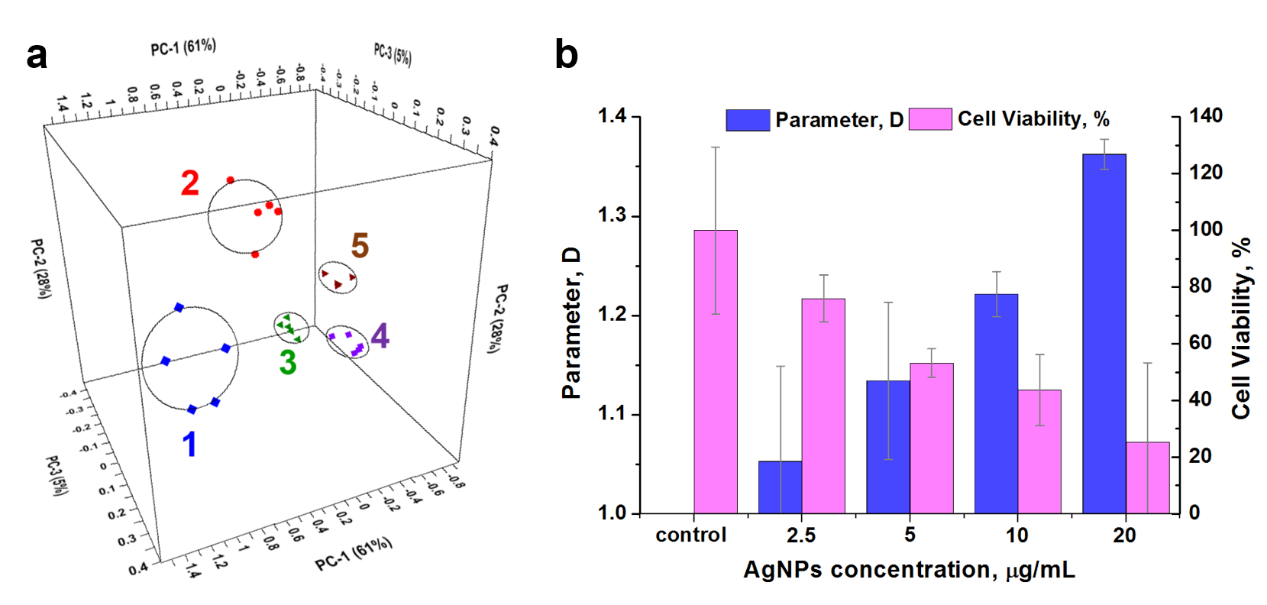


**Figure S8.** **a.** The PCA results of AgNPs antibacterial experiment. 1-5 principal components are from the sample incubated with different AgNPs concentration at 0, 2.5, 5, 10 and 20μg/mL for 2h. Figure **b** shows the relationship between AgNPs concentration and parameter D as well as the cell viability rates of *E.coli*.


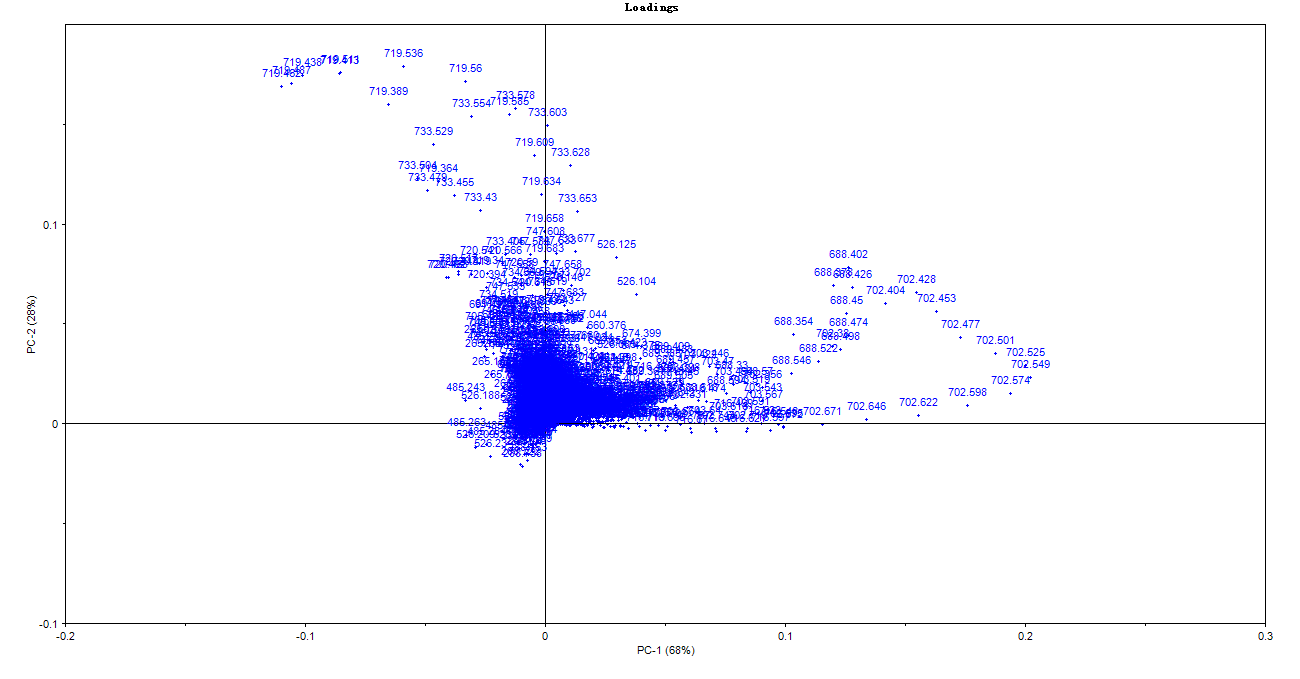


**Figure S9**. The PCA loading plot of GO concentration experiment, the spot location in PCA loading plot represents its contribution to the separation of principal components in PCA scores plot.


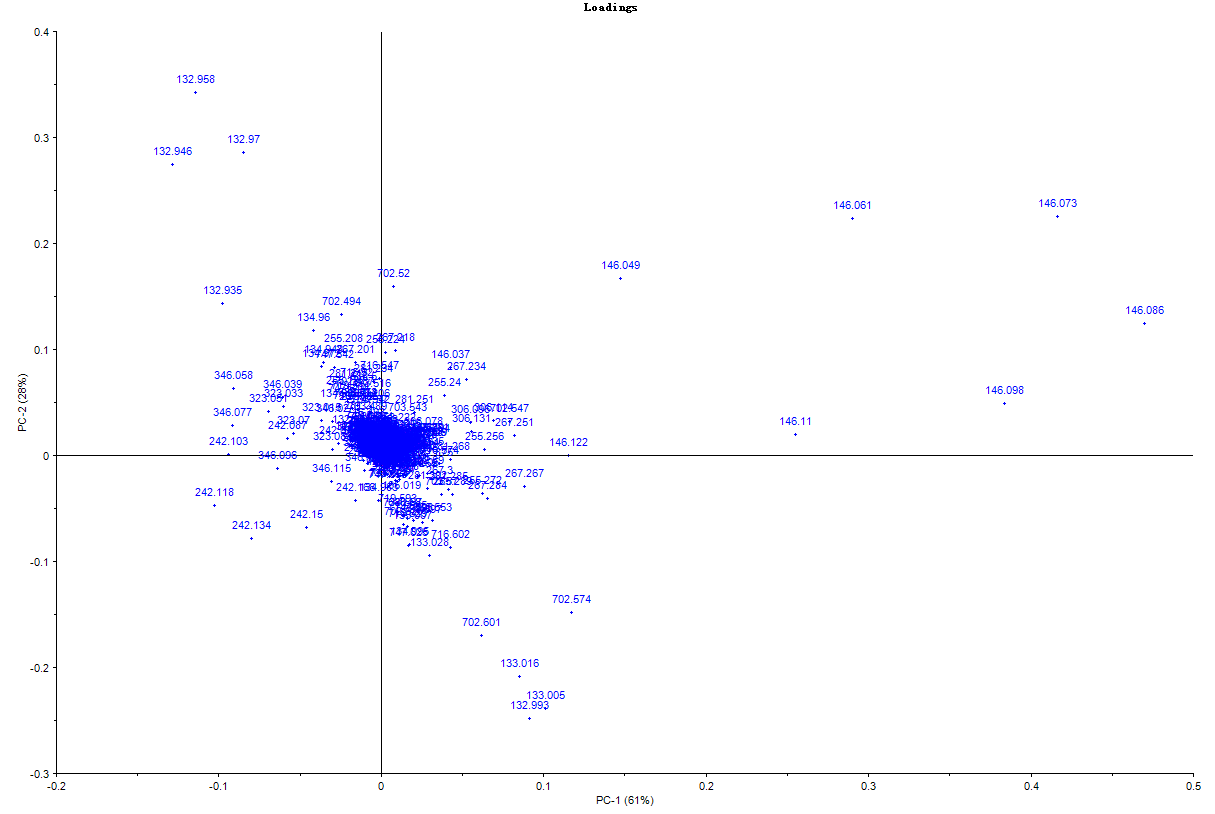


**Figure S10**. The PCA loadings plot of AgNPs incubation experiment,


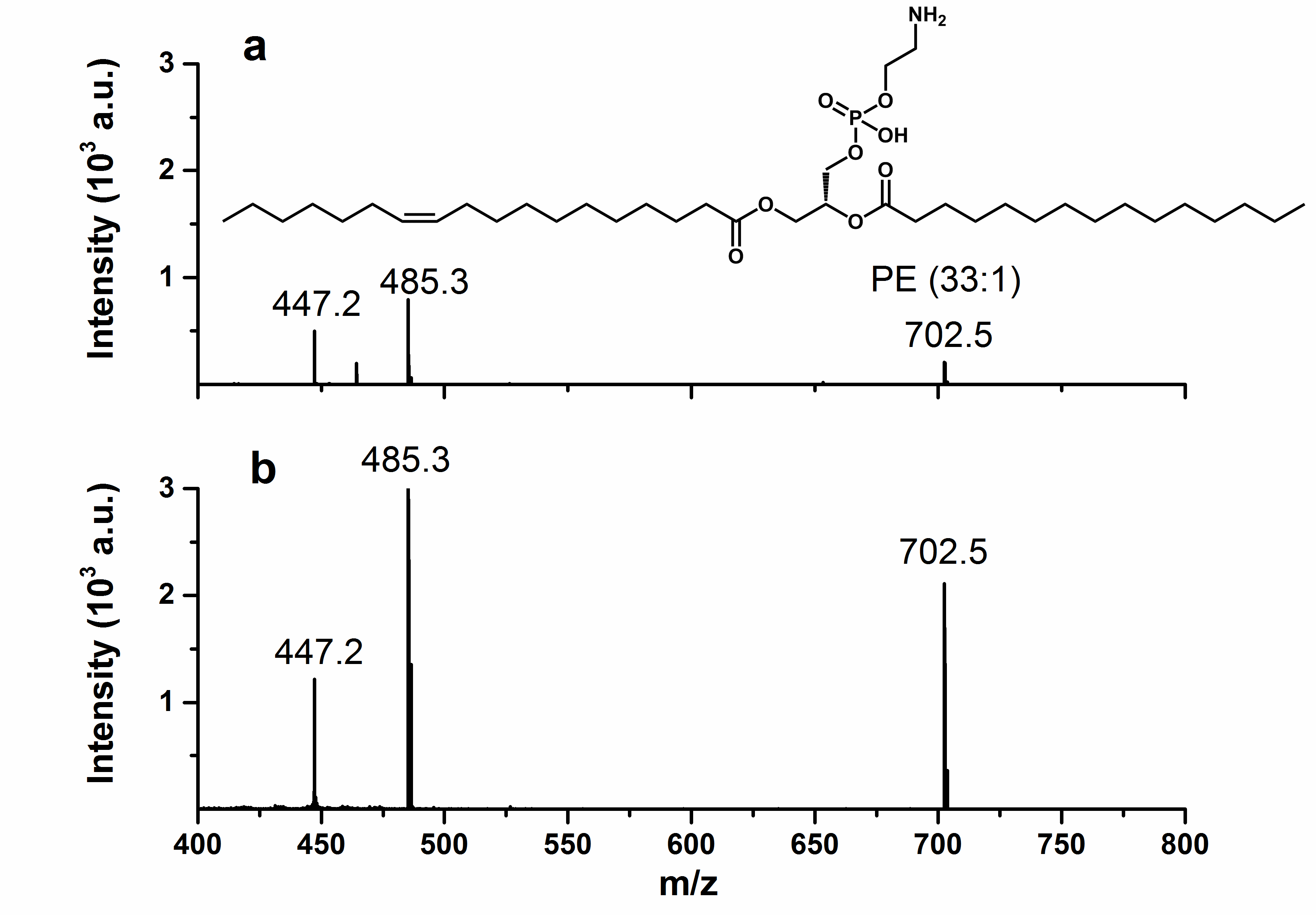


**Figure S11**. The mass spectra of supernatant solution after the microbe was collected by centrifugation (a) control (b) incubated with 80μg/mL GO.


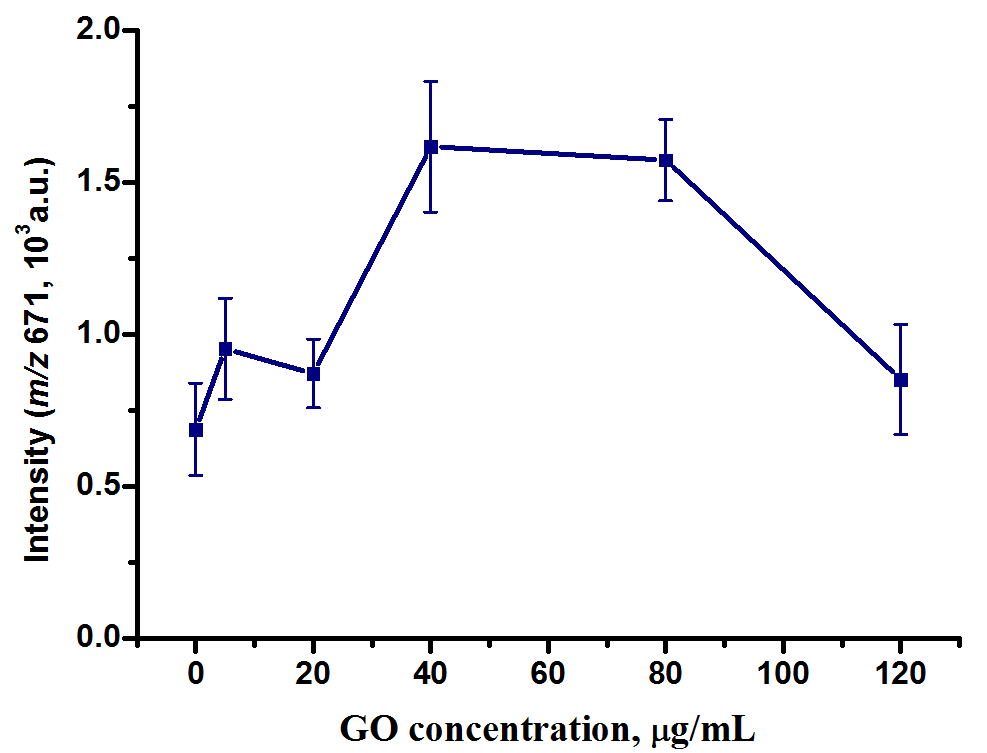


**Figure S12.** The intensity variation of PA (30:2) (*m/z* 671) with GO incubation concentration increasing.

**Table S1**. The substances in Figure1 mass spectra which have been identified by database searching and high resolution mass spectrometry or tandem mass spectrometry.

| **Name** | **Chemical Formula** | **Form** | **m/z (TOF)** | **HR m/z**  **(FT-ICR)** | **theoretical m/z** | **Delta m/z** | **Deviation**  **(ppm)** |
| --- | --- | --- | --- | --- | --- | --- | --- |
| **Sodium** | **Na** | **M+2Cl** | **92.925** | **-** | **92.926926** |  |  |
| **Phophate** | **H3PO4** | **M-H/M+Cl** | **96.966/132.955** | **-** | **96.968522** |  |  |
| **Hypotaurine** | **C2H7NO2S** | **M-H** | **108.001** | **-** | **108.012473** |  |  |
| **Potassium** | **K** | **M+Cl** | **108.916** | **-** | **108.901961** |  |  |
| **L-Glutamic acid** | **C5H9NO4** | **M-H** | **146.018** | **-** | **146.04588** |  |  |
| **Carbamoylphosphate** | **CH4NO5P** | **M-H** | **139.985** | **-** | **139.975433** |  |  |
| **Citric acid** | **C6H8O7** | **M-H** | **190.997** | **-** | **191.019727** |  |  |
| **2-Hydroxy-6-ketononadienedicarboxylate** | **C9H8O6** | **M-H** | **211.051** | **-** | **211.023714** |  |  |
| **Hexyl octanoate** | **C14H28O2** | **M-H** | **227.179** | **227.20171** | **227.201654** | **0.000056** | **0.2** |
| **Palmitoleic acid** | **C16H30O2** | **M-H** | **253.21** | **253.21742** | **253.217304** | **0.000116** | **0.5** |
| **Palmitic acid** | **C16H32O2** | **M-H** | **255.217** | **255.233** | **255.232954** | **0.000046** | **0.2** |
| **Heptadecenoic acid** | **C17H32O2** | **M-H** | **267.218** | **267.23299** | **267.232954** | **0.000036** | **0.1** |
| **Vaccenic acid** | **C18H34O2** | **M-H** | **281.184** | **281.24854** | **281.248604** | **0.000064** | **0.2** |
| **Glutathione** | **C10H17N3O6S** | **M-H** | **306.067** | **306.0769** | **306.07653** | **0.00037** | **1.2** |
| **Dodecylbenzenesulfonic acid** | **C18H30O3S** | **M-H** | **325.122** | **325.18438** | **325.18429** | **0.00009** | **0.3** |
| **Dihydrotestosterone sulfate** | **C19H30O5S** | **M-H20-H** | **351.095** | **351.16168** | **351.163005** | **0.001325** | **3.8** |
| **Lyso-phosphatidylethanolamine(16:0)** | **C19H38NO7P** | **M-H** | **452.221** | **452.278263** | **453.285539** | **0.000167** | **0.4** |
| **Lyso-phosphatidylethanolamine(18:1)** | **C24H46O9P** | **M-H** | **509.181** | **509.28883** | **509.2885** | **0.0003** | **0.6** |
| **Phosphatidylethanolamine(28:0)** | **C33H65NO8P** | **M-H** | **634.393** | **634.44534** | **634.4453** | **0** | **0.0** |
| **Glycerol 1-(9Z-octadecenoate) 2-tetradecanoate 3-phosphate** | **C35H67O8P** | **M-H** | **645.387** | **645.45008** | **646.457356** | **0.00011** | **0.2** |
| **Phosphatidylethanolamine(29:0)** | **C35H68O10P** | **M-H** | **648.407** | **648.460979** | **649.468255** | **0.000859** | **1.3** |
| **Phosphatidylethanolamine(30:0)** | **C36H70O12P** | **M-H** | **662.426** | **662.476629** | **663.483905** | **0.000261** | **0.4** |
| **Phosphatidylglycerol(28:0)** | **C34H66O10P** | **M-H** | **665.388** | **665.44013** | **665.4399** | **0.0002** | **0.3** |
| **Phosphatidic acid(30:2)** | **C33H61O8P** | **M-H** | **671.412** | **671.46584** | **671.46573** | **0.00011** | **0.2** |
| **Phosphatidylethanolamine(31:1)** | **C36H69NO8P** | **M-H** | **674.419** | **674.47622** | **674.4766** | **0.0004** | **0.6** |
| **Phosphatidylethanolamine(31:0)** | **C36H71NO8P** | **M-H** | **676.438** | **676.49296** | **676.4923** | **0.0006** | **0.9** |
| **Sphingomyelin(d18:0/16:0)** | **C39H82N2O6P** | **M-H20-H** | **686.405** | **686.56682** | **686.57266** | **0.00584** | **8.5** |
| **Phosphatidylethanolamine(32:1)** | **C37H71NO8P** | **M-H** | **688.454** | **688.49223** | **688.4923** | **0.0001** | **0.1** |
| **Phosphatidylethanolamine(32:0)** | **C37H73NO8P** | **M-H** | **690.49** | **690.5085** | **690.5079** | **0.0006** | **0.9** |
| **Phosphatidylethanolamine(30:1)** | **C36H68O10P** | **M-H** | **691.463** | **691.45642** | **691.4555** | **0.0009** | **1.3** |
| **Phosphatidylethanolamine(33:2)** | **C38H71NO8P** | **M-H** | **700.492** | **700.49212** | **700.4923** | **0.0002** | **0.3** |
| **Phosphatidylethanolamine(33:1)** | **C38H73NO8P** | **M-H** | **702.519** | **702.50846** | **702.5079** | **0.0005** | **0.7** |
| **Phosphatidylethanolamine(31:1)** | **C37H70O10P** | **M-H** | **705.54** | **705.47215** | **705.4712** | **0.0009** | **1.3** |
| **Phosphatidylethanolamine(31:0)** | **C37H72O10P** | **M-H** | **707.549** | **707.4874** | **707.4868** | **0.0006** | **0.8** |
| **Phosphatidylethanolamine(34:2)** | **C39H73NO8P** | **M-H** | **714.507** | **714.50815** | **714.5079** | **0.0002** | **0.3** |
| **Phosphatidylglycerol(32:1)** | **C38H72O10P** | **M-H** | **719.565** | **719.48747** | **719.4868** | **0.0006** | **0.8** |
| **Phosphatidylglycerol(32:0)** | **C38H74O10P** | **M-H** | **721.597** | **721.50339** | **721.5025** | **0.0009** | **1.2** |
| **Phosphatidylethanolamine(35:2)** | **C40H75NO8P** | **M-H** | **728.529** | **728.52409** | **728.5236** | **0.0005** | **0.7** |
| **Phosphatidylethanolamine(35:1)** | **C40H77NO8P** | **M-H** | **730.539** | **730.5399** | **730.5392** | **0.0007** | **1.0** |
| **Phosphatidylglycerol(33:1)** | **C39H74O10P** | **M-H** | **733.592** | **733.50299** | **733.5025** | **0.0005** | **0.7** |
| **Phosphatidylethanolamine(36:2)** | **C41H77NO8P** | **M-H** | **742.543** | **742.53969** | **742.5392** | **0.0005** | **0.7** |
| **Phosphatidylethanolamine(36:1)** | **C41H79NO8P** | **M-H** | **744.661** | **744.55616** | **744.5549** | **0.0012** | **1.6** |
| **Phosphatidylglycerol(34:2)** | **C40H74O10P** | **M-H** | **745.599** | **745.50301** | **745.5025** | **0.0005** | **0.7** |
| **Phosphatidylglycerol(34:1)** | **C40H76O10P** | **M-H** | **747.614** | **747.51873** | **747.5181** | **0.0006** | **0.8** |
| **Phosphatidylethanolamine(37:2)** | **C42H79NO8P** | **M-H** | **756.607** | **756.5552** | **756.5549** | **0.0003** | **0.4** |
| **Phosphatidylglycerol(35:2)** | **C41H76O10P** | **M-H** | **759.614** | **759.51865** | **759.5181** | **0.0005** | **0.7** |
| **Phosphatidylethanolamine(P-36:4/20:4)** | **C41H74NO7P** | **M+K-2H** | **760.56** | **760.46754** | **760.468896** | **0.001356** | **1.8** |
| **Phosphatidylglycerol(35:1)** | **C41H78O10P** | **M-H** | **761.624** | **761.53441** | **761.5338** | **0.0006** | **0.8** |
| **Phosphatidylglycerol(36:2)** | **C42H78O10P** | **M-H** | **773.621** | **773.53422** | **773.5338** | **0.0004** | **0.5** |
| **Phosphatidylglycerol(45:8)** | **C51H84O10P** | **M-H** | **887.656** | **887.57981** | **887.5807** | **0.0009** | **1.0** |

**Table S2**. The substances are concluded of which intensity changed obviously as E.coli incubated with 80 μg/mL GO. Parallel 1 and 2 are the results from twice experiments. When the intensity ratio is less than 1, it means the content of the related substance decrease.

| **Metabolites** | ***m/z*** | **Intensity Ratio** | |
| --- | --- | --- | --- |
|  |  | **parallel 1** | **parallel 2** |
| **Palmitoleic acid** | **253.21** | **0.63** | **0.85** |
| **Palmitic acid** | **255.22** | **0.42** | **0.22** |
| **Heptadecenoic acid** | **267.22** | **0.39** | **0.23** |
| **Vaccenic acid** | **281.19** | **0.47** | **0.24** |
| **Dodecylbenzenesulfonic acid** | **325.12** | **1.81** | **4.43** |
